# Inward versus reward: white matter pathways in extraversion

**DOI:** 10.1017/pen.2019.6

**Published:** 2019-09-16

**Authors:** R. Leshem, P. Paoletti, C. Piervincenzi, F. Carducci, C. A. Mallio, Y. Errante, C. C. Quattrocchi, T. Dotan Ben-Soussan

**Affiliations:** 1 Department of Criminology, Bar-Ilan University, Ramat-Gan, Israel; 2 Research Institute for Neuroscience, Education and Didactics, Patrizio Paoletti Foundation, Neuroscientific Research Unit, Assisi, Italy; 3 Departmental Faculty of Medicine and Surgery, Università “Campus Bio-Medico di Roma”, Rome, Italy; 4 Department of Physiology and Pharmacology, Neuroimaging Laboratory, Sapienza University, Rome, Italy

**Keywords:** diffusion tensor imaging, white matter integrity, inferior fronto-occipital fasciculus (IFOF), extraversion, personality

## Abstract

The trait of extraversion is one of the longest-standing domains that captures the social dimension of personality and can potentially explain the covariation of a wide variety of behaviors. To date, there is a growing recognition that human behavior should be specified not only through the psychological mechanisms underlying each trait but also through their underlying neurobehavioral systems. While imaging studies have revealed important initial insights into the structural and functional neural correlates of extraversion, current knowledge about the relationships between extraversion and brain structures is still rather limited, especially with regard to the relationship between extraversion and white matter (WM). In this study, we aimed to investigate WM microstructure in extraversion in greater depth. Thirty-five healthy volunteers (21 women; mean age 35) underwent magnetic resonance imaging, as a part of a larger project aimed at investigating the longitudinal effect of motor training. WM integrity was assessed using the diffusion tensor imaging technique combining multiple diffusion tensor measures. Extraversion was assessed by the Eysenck Personality Questionnaire-Revised. Voxelwise correlation analyses between fractional anisotropy, axial diffusivities, and radial diffusivities maps and extraversion score showed decreased connectivity in the right inferior fronto-occipital fasciculus and forceps major among individuals who had high extraversion ratings. In conclusion, individual differences in extraversion may reflect differential organization of the WM tracts connecting frontal cortex, temporal, and occipital areas, which are related to socioemotional and control functions.

Personality traits involve individual tendencies to express specific patterns of cognition, emotion, and behavior (Xu & Potenza, 2012). The attitudes people have toward their environments, as well as their dispositions, personal preferences, and dislikes, all help determine their everyday actions, subjective well-being, mental and physical health, quality of relationships with peers and family members, and occupational performance and satisfaction (Mitchell & Kumari, 2016; Xu & Potenza, 2012). A large body of evidence describes human personality with respect to five core dimensions: extraversion, neuroticism, agreeableness, conscientiousness, and openness/intellect (Costa & McCrae, 1992; Wright et al., 2006).

The extraversion dimension, which involves individual differences in social behavior and socioemotional functioning, is considered one of the fundamental dimensions of personality (Costa & McCrae, 1992; Wilt & Revelle, 2009). As such, extraversion can potentially explain the covariation of a wide variety of behaviors and predict risk for and resilience to different forms of psychopathology, such as affective disorders (Wilt & Revelle, 2009; Wright et al., 2006) and addictive behaviors (Munafo & Black, 2007; Trémeau et al., 2003). Up to half of the variability in extraversion is believed to be heritable, suggesting that interindividual differences in this trait have biological bases in the brain (Kanai & Rees, 2011). As such, in personality neuroscience, there is a growing recognition that human behavior should be specified not only through the psychological mechanisms underlying each trait but also through the neurobehavioral systems that are linked to those psychological mechanisms (Depue & Collins, 1999; DeYoung et al., 2010; Mitchell & Kumari, 2016).

Hans Eysenck (1967) was one of the first investigators to explore and develop a neurobiological model for personality. His explanation, which attempted to define neurophysiological sources of personality, uniquely addressed two major personality dimensions, extraversion and neuroticism, in terms of individual differences in central nervous system functions (Eysenck, 1967). According to Eysenck’s model, individuals who are high in extraversion have lower cortical arousal and therefore require external stimulation. Those who score low on measures of extraversion (i.e., introverts) have lower response thresholds and are consequently more cortically aroused than those with high extraversion scores. The arousal theory generated a wide range of studies, some of which showed moderate support at most for the relationship between extraversion and regional arousal (Gao et al., 2013; Küssner, 2017; Wilt & Revelle, 2009), suggesting that this relationship is not as simple as originally proposed by Eysenck (for further discussion, see Zuckerman & Glicksohn, 2016). Gray (1970) further examined the physiology of extraversion using an animal model. He argued that extraversion–introversion reflect motivational systems that evolved to facilitate adaptation to classes of stimuli associated with positive and negative reinforcement. In accordance, differences in extraversion have been shown to be related to structural and functional differences in brain structures implicated in emotion and motivation. The brain structures most often studied in relation to extraversion are the prefrontal cortex (PFC), orbitofrontal cortex (OFC) (Depue & Collins, 1999; DeYoung et al., 2010; Xu & Potenza, 2012), anterior cingulated cortex (ACC), parietal cortex, striatum, middle temporal gyrus (MTG), and amygdala (Canli, 2004; Canli et al., 2001; Eisenberger, Lieberman, & Satpute, 2005; Haas, Omura, Amin, Constable, & Canli, 2006; Koelsch, Skouras, & Jentschke, 2013). These structures are all involved in socioemotional processes and have been tied specifically to the regulation of behavior and emotion (Leshem, 2016).

Functional connectivity studies at rest (in the absence of presented stimuli or task instructions) as well as task-based studies show anatomic variations in different brain areas, with a variety of both positive and negative correlations identified between extraversion and neuroanatomic measures in several regions. Some studies reported a positive association between extraversion and gray matter (GM) in the OFC and sub-cortical regions related to emotional processing (Cremers et al., 2011; DeYoung et al., 2010), whereas others have found no significant correlation between extraversion and GM in the OFC and amygdala (Wright et al., 2006). Additional studies have reported negative associations with GM in frontal-subcortical circuits, which are known to contribute to cognitive control and emotional functions (e.g., ventrolateral PFC, superior/middle frontal gyrus, MTG, and amygdala) (Blankstein, Chen, Mincic, McGrath, & Davis, 2009; Coutinho, Sampaio, Ferreira, Soares, & Gonçalves, 2013; Gao et al., 2013; Lu et al., 2014; Omura, Constable, & Canli, 2005).

The inconsistencies between studies reflect the instability of results in personality neuroscience, in general, perhaps because the relevant biological data depend on responses to different situations and to individual differences in extraversion-related traits (see Zuckerman & Glicksohn, 2016). Analysis methods are another potential source of variance (Privado et al., 2017; Wright et al., 2006).

While the studies presented above have revealed important initial insights into the structural and functional neural correlates of extraversion, current knowledge about the relationships between extraversion and brain function and structures is still rather limited (Mitchell & Kumari, 2016), especially with regard to the relationship between extraversion and white matter (WM). WM mediates communications in the brain and is critical for the integrity of the brain functions underlying emotional and cognitive processes (Privado et al., 2017; Xu et al., 2012).

The current study aimed to investigate the relationship between extraversion and WM integrity using the diffusion tensor imaging (DTI) technique. DTI assesses WM integrity by measuring directional water diffusion (Basser, Mattiello, & LeBihan, 1994; Pierpaoli, Jezzard, Basser, Barnett, & DiChiro, 1996). The DTI technique is sensitive to the magnitude and orientation of water diffusion throughout brain tissue and exploits this information to calculate several diffusion parameters through a tensor model. The most commonly used DTI parameter is the fractional anisotropy (FA). FA value designates the degree of anisotropy (a measure of how highly oriented substructures are within a volume) of a diffusion process. However, FA may not be sufficient for the investigation of specific axonal density and diameter and/or myelination (Jones, Knösche, & Turner, 2013). FA values are thought to reflect WM integrity, as a result of greater intravoxel coherence of fiber orientation, axon density and diameter, and/or myelination (Beaulieu, 2002; Caminiti et al., 2013; Sen & Basser, 2005). Yet, many factors may affect water anisotropy and consequently the FA value of a given fiber (e.g., myelin, cell membrane, axonal diameter, and density), and each of these factors can influence axonal conduction velocity (Assaf & Pasternak, 2008; Tavor et al., 2014). Thus, for example, different combinations of axial diffusivities (AD) and radial diffusivities (RD) can give rise to identical FA values. The relationship between AD and RD (degree of anisotropy) can be expressed by FA (Jones et al., 2013). As such, increasing axonal density, reducing axonal caliber, and increasing the degree of myelination should all lead to reduced RD and therefore elevated FA (Jones et al., 2013). Since AD is thought to reflect axonal integrity and RD seems to be sensitive to myelination, joint use of the two indices makes it possible to capture different aspects of WM microstructure separately and to provide additional important information regarding WM organization (Jiang et al., 2017). In the current study, multiple diffusion tensor measures (i.e., FA, AD, and RD) were employed to understand WM microstructure in extraversion in greater depth.

Recently, DTI has been used to assess correlations between the WM tracts interconnecting different brain regions (e.g., hippocampus, amygdala, striatum, and PFC) and behaviors related to selected personality traits, such as novelty-seeking, reward dependence, and creativity (Cohen, Schoene-Bake, Elger, & Weber, 2009; Jung, Grazioplene, Caprihan, Chavez, & Haier, 2010; Takeuchi et al., 2010; Xu et al., 2012). A study by Xu and Potenza (2012) assessed the relationships between WM integrity and personality traits among 51 healthy participants using FA and MD DTI measures and the revised NEO Personality Inventory. The researchers found no correlations between extraversion scores and the integrity of the WM tracts interconnecting the PFC, parietal cortex, and striatum. Also, functional connectivity (resting-state and task-based fMRI) studies have found positive correlations between extraversion and functional connectivity between the ACC, inferior parietal lobe, and PFC (DeYoung et al., 2010; Haas et al., 2006; Mobbs, Hagan, Azim, Menon, & Reiss, 2005). These positive correlations reflect high activity within the reward circuitry in response to rewarding stimuli in cases of high trait extraversion (Aghajani et al., 2014). In their study, Privado et al. (2017) analyzed relationships of both GM and WM to individual differences in the personality traits designated by the Five-Factor Model. Their results showed significant associations between extraversion and occipital cortical surface area variations. They did not find significant correlations between extraversion and selected WM tracts connecting anterior with posterior regions, in both hemispheres, or frontal-temporal regions. In addition, Privado et al. found that the variations in discrete GM clusters were associated with temperamental traits (extraversion), whereas long-distance structural connections were related to openness, the personality dimension that has been associated with high-level cognitive processes.

To date, most of the MRI studies examining personality traits, including extraversion, have focused on measures of functional and structural connectivity that indicate increased/decreased involvement of brain regions associated with cognitive and emotional processing. To the best of our knowledge, little work has examined WM pathways in extraversion (e.g., Privado et al., 2017; Xu & Potenza, 2012). In the present study, we aimed to further explore possible correlations between extraversion FA, AD, and RD. Given the core features of extraversion (DeYoung et al., 2010), we predicted that low FA/AD and high RD values in WM pathways that interconnect regions are associated with extraversion-related tendencies (e.g., sociability, novelty and sensation seeking, reward sensitivity, and positive emotion).

## 1. Materials and methods

### 1.1 Participants and design

The participants belong to a larger study comprising three time points (T0, T1, and T2, Piervincenzi et al., 2017). We recruited 50 healthy volunteers. Following the inclusion and exclusion criteria (see Piervincenzi et al., 2017), four participants were excluded due to the presence of WM lesion, three participants because they were left-handed, six participants because of MRI incomplete protocol, and two participants because of lack in complying motor exercise. Thus, the analyses were conducted in a group of 35 healthy right-handed participants (21 women and 14 men, mean age ± SD: 35 ± 5 and 36 ± 5 years, respectively). The correlation between FA, AD and RD and the Eysenck Personality Questionnaire subscales scores were conducted only at T2, after 12 weeks of Quadrato Motor Training (QMT). QMT is a whole-body mindful movement practice created by Patrizio Paoletti, in which the participant is asked to move within a 50 by 50 cm^2^.

### 1.2 Ethics statement

All procedures were explained to participants, verifying sufficient understanding, and written informed consent was obtained in accordance with the Declaration of Helsinki. The study was approved by the ethical committee of the Università Campus Bio-Medico di Roma, Rome, Italy.

*The Eysenck Personality Questionnaire-Revised* (EPQ-R, Eysenck & Eysenck, 1991). This is a 106-item forced-choice personality questionnaire. Here, we focused on three subscales, namely: Extraversion (*E*) subscale = 23 items (item example: “Do you have many friends?”); Psychoticism (*P*) subscale = 23 items (item example: “Do you enjoy to insult people who you love?”); and Neuroticism (*N*) subscale = 24 items (item example: “Have you often felt guilty?”); on which participants answered choosing between YES and NO. Reliability coefficients for the Extraversion and Neuroticism scales ranged from 0.85 to 0.90 (Eysenck & Eysenck, 1991). The scale was translated from English to Italian by Dazzi (2011) with good reliability for *E*, *P*, and *N* (0.78, 0.67, and 0.85, respectively). In the current study, the alpha coefficients were .68, .67, and .79, respectively.

### 1.3 MRI data acquisition

Imaging data were acquired using a Siemens 1.5-T MAGNETOM Avanto (Siemens, Erlangen, Germany) whole body scanner equipped with a 12-element designed Head Matrix coil, as part of the standard system configuration. Diffusion-weighted images (DWIs) were acquired using an axial pulsed-gradient spin-echo echo-planar sequence (7600/103; 38 sections; section thickness, 3.0 mm with no intersection gap), with diffusion-encoding gradients applied in 12 noncollinear directions (*b* factor 0 and 1000 sec/mm^2^; number of acquired signals, four). A 2D fluid-attenuated inversion recovery (FLAIR) T2-weighted scan was also used to exclude the presence of small vessel ischemic disease and other supra- or infra-tentorial brain lesions (Repetition Time [TR] = 11 460 ms, Echo Time [TE] = 102 ms, Inversion Time [TI] = 2360 ms, Field of View [FOV] = 280 mm × 330 mm, Number of Excitations [NEX] = 2, matrix = 248 × 320, 1.00 × 1.00 mm^2^ in-plane resolution, horizontal slices with a slice thickness of 3.0 mm and no gap).

### 1.4 MRI data analysis

To avoid a type I error induced by the effect of WM hyperintensities on brain connectivity results, two expert radiologist (CCQ, YE) examined all MRIs. Participants were excluded when more than three lesions with a maximum diameter of 5 mm were detected in the subcortical or periventricular WM on axial FLAIR images (Quattrocchi et al., 2015).

#### 1.4.1 Preprocessing of diffusion data

All DWIs were visually inspected for artifacts and preprocessed using different tools from FDT [FMRIB Diffusion Toolbox, part of FSL (FMRIB’s Software Library v.5.0.8, https://www.fmrib.ox.ac.uk/fsl/); (Smith et al., 2004)]. Images were corrected for eddy current distortion and head motion using a 12 parameter affine registration to the first no-diffusion-weighted volume of each participant, and the gradient directions were rotated accordingly (Leemans & Jones, 2009). Corrected images were skull-stripped using Brain Extraction Tool (Smith, 2002). Diffusion tensor images were then generated for each participant and each time point using the Diffusion Tensor Imaging ToolKit software package (DTI-TK, https://dti-tk.sourceforge.net/pmwiki/pmwiki.php) (Zhang, Yushkevich, Alexander, & Gee, 2006).

An unbiased longitudinal analysis approach was chosen for the registration of DTI data (Keihaninejad et al., 2013) using DTI-TK, which applies a registration algorithm that leverages the full diffusion tensor information to drive the registration, improving the alignment of WM structures (Wang et al., 2011). At the end of the registration procedure, each participant’s DTI data were normalized to the ICBM-152 template (Zhang, Peng, Dawe, & Arfanakis, 2011), and FA maps were generated for each participant using the normalized tensor images (for details on the DTI data analysis, see Piervincenzi et al., 2017).

FA data from each participant were further analyzed using selected modules of the Tract-Based Spatial Statistics (Smith et al., 2006) toolbox, available in FSL. The mean FA image was created and thinned to create a mean FA skeleton, which represents the centers of all tracts common to the group. Each participant’s FA image was then projected onto this common skeleton to minimize any residual misalignment of tracts.

Randomize tool (5000 permutations) was used to examine the statistical correlation between FA maps at T2 and EPQ’s subscale (i.e., *E*, *P*, and *N*) scores. The statistical threshold was established with a family-wise error-corrected *p* value (*p*FWE) < .05 with multiple comparison corrections using threshold-free cluster enhancement (TFCE) (Smith & Nichols, 2009). All the results were anatomically localized using the Johns Hopkins University (JHU) ICBM-DTI-81 White-Matter Labels and the JHU White-Matter Tractography atlases included in the FSL distribution (https://www.fmrib.ox.ac.uk/fsl/fslwiki/Atlases).

#### 1.4.2 Statistical analysis

Voxelwise correlation analyses were conducted to assess the relationship between FA values and EPQ subscale scores.

## 2. Results

A voxelwise correlation analyses between FA, AD, and RD values and EPQ subscale scores showed that extraversion score was negatively correlated with FA/AD values and positively correlated with RD value in WM within the right inferior fronto-occipital fasciculus (IFOF) and forceps major (posterior).

**Figure 1.**
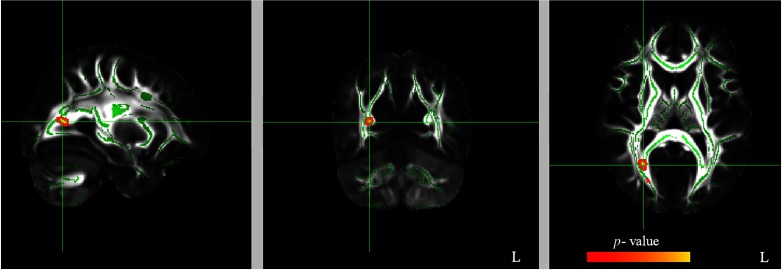
Significant negative correlation (*p*FWE < .05 TFCE-corrected) between FA values and Extraversion (red to yellow color). The study-specific FA skeleton, representing the centers of principal WM tracts, is displayed in green, overlaid on the mean FA map (for methodological details, see also Piervincenzi et al., 2017).

Results of voxelwise correlation analyses between FA values and EPQ subscale scores are shown in Table 1. A strong negative correlation (*p*FWE < .05 TFCE-corrected) was found between FA values and Extraversion (see Table 1, Figure 1). No significant correlation was found between FA values and Neuroticism or Psychoticism.

**Table 1.** Results of voxelwise correlation analyses between FA, RD, and AD values at T2 (after 12 weeks of QMT) and EPQ’s subscale scores. Negative correlations (*p*FWE < .05 TFCE-corrected) were found between FA/AD values and Extraversion, whereas a positive correlation (*p*FWE < .05 TFCE-corrected) was found between RD values and Extraversion

Cluster size	*T*	*p*	MNI coordinates			Pathway/region
			*x*	*y*	*z*	
FA – Extraversion ↓						
27	6.16	.004	29	−65	11	Forceps major, inferior fronto-occipital fasciculus R
RD – Extraversion ↑						
22	4.24	.001	29	−66	12	Forceps major, inferior fronto-occipital fasciculus R
AD – Extraversion ↓						
15	3.73	.003	29	−66	13	Forceps major, inferior fronto-occipital fasciculus R

**Figure 2.**
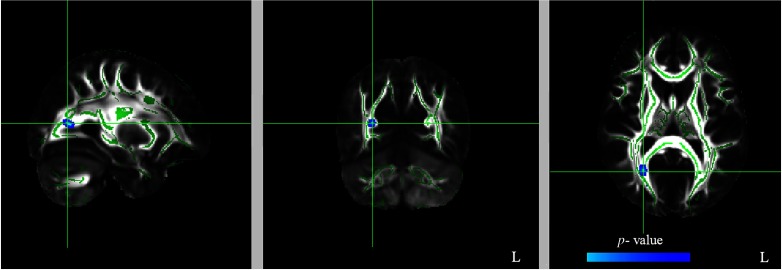
Significant positive correlation (*p*FWE < .05 TFCE-corrected) between RD values and Extraversion (blue to light blue color). See Figure 1 for additional details.

**Figure 3.**
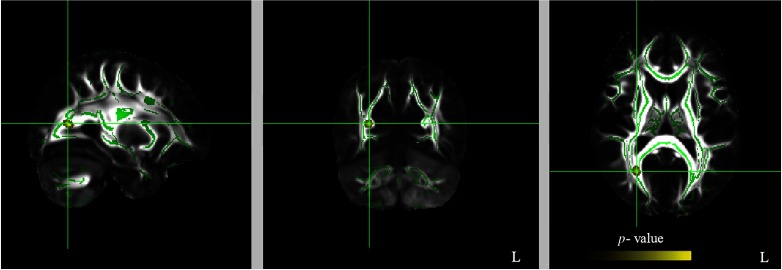
Significant negative correlation (*p*FWE < .05 TFCE-corrected) between AD values and Extraversion (dark yellow to yellow color). See Figure 1 for additional details.

Since a strong negative correlation (*p*FWE < .05 TFCE-corrected) was found between FA values and *E* subscale, we then examined possible correlation between Extraversion and RD (Table 1, Figure 2) as well as AD (Table 1, Figure 3) values.

## 3. Discussion

This study examined the correlations between the integrity of WM tracts and extraversion, using FA, AD, and RD indices. In accordance with our prediction, we found a correlation between extraversion and specific fibers within WM tracts. More specifically, extraversion score was negatively correlated with FA/AD values and positively correlated with RD value, suggesting that high extraversion score was associated with decreased organization of WM tracts (FA) and demyelination (RD) in WM within the right IFOF and forceps major (posterior). These WM fibers interconnect brain regions that are implicated in emotional processing and cognitive control functions related to extraversion.

The IFOF is a large WM tract connecting the frontal (infero-lateral and dorso-lateral frontal), temporal, and occipital lobes and constitutes one of the major efferent and afferent neuronal projections to the frontal lobes. It runs from the lateral aspect of the frontal lobe, passing through the extreme and external capsules, as well as the temporal lobe stem, before radiating into the posterior temporal and occipital cortex (Catani, Howard, Pajevic, & Jones, 2002). WM in these association fibers mediates communications in brain areas that are active in controlling emotion and behavior (Kim & Whalen, 2009). Furthermore, IFOF is involved in insula connectivity, which is believed to play an important role in emotional regulation and cognition (Catani et al., 2002).

The fibers of the posterior portion of the corpus callosum (CC) or the splenium form the forceps major (Catani et al., 2002), which interconnect the two hemispheres and include fibers with projection zones in temporal, parietal, and occipital lobes (Witelson, 1989). In general, the CC connects the two cerebral hemispheres and is believed to play an important role in supporting sensory-motor functional integration, attention, language, interhemispheric transfer of associative learning, and emotional regulation and in understanding social situations and adopting appropriate social behavior (Jiang et al., 2017). Studies of callosotomies and research on animals indicate that disruption of commissural tracts can cause imbalances in interhemispheric communication (see Bloom & Hynd, 2005).

The IFOF and forceps major WM tracts hold the most prominent fibers running between the cognitive control and emotional systems in the brain and are responsible for effective communication between them. As evidenced by neurodevelopmental studies, improved cognitive and emotional functions are thought to involve maturation of functional connections subserved by ongoing myelination, which allows more rapid and efficient communication between neurons (Spear, 2000). Specifically, the increasing organization of WM from childhood to early adulthood, particularly in the prefrontal area but also in the parietal, occipital, and temporal lobes, corresponds to the development of cognitive control. Furthermore, the interaction of PFC with other functionally specialized brain regions through WM has been increasingly recognized as critical for these integrative brain functions (Clark, Thatcher, & Tapert, 2008; Luna, Paulsen, Padmanabhan, & Geier, 2013; Olson et al., 2009).

In the current study, we found decreased connectivity (low FA/AD together with high RD) values in the right IFOF and in the forceps major among individuals who had high extraversion ratings. Notably, the negative correlation between extraversion and WM integrity in right IFOF, which interconnects frontal-temporal-occipital regions, suggests that lateralization of these regions may play a role in expressions of extraversion. Right lateralization has been associated with an increased tendency toward negative emotions and withdrawal tendencies (Davidson, 1992; Davidson, Ekman, Saron, Senulis, & Friesen, 1990), which comprise an introversion-associated dimension (Pang et al., 2017). It may be, then, that the decreasing organization of WM tracts, as reflected in low FA/AD and high RD (demyelination), in these pathways results in disinhibition, approach tendencies, and emotionally oriented behaviors (especially positive ones), many of which mediate extraversion. Extraversion is considered a higher-order trait that encompasses a set of correlated lower-order traits (Depue & Collins, 1999), which include behavioral and emotional characteristics such as social dominance, positive emotions, sociability, achievement, reward sensitivity, novelty seeking, and motor activity (Costa & McCrae, 1992; Depue & Collins, 1999; DeYoung et al., 2010; Eysenck & Eysenck, 1985). Furthermore, in their neurobehavioral model of extraversion, Depue and Collins (1999) defined extraversion as having two characteristics: (1) *interpersonal engagement*, which consists of affiliation (enjoying and valuing close interpersonal bonds, being warm and affectionate) and agency (being socially dominant, enjoying leadership roles, being assertive, being exhibitionistic, and having a sense of potency in accomplishing goals) and (2) *impulsivity*, which emerges from the interaction of extraversion and a second independent trait (constraint).

We speculated that the aforementioned individual differences in extraversion might reflect decreasing organization of the WM tracts connecting frontal cortex, temporal, and occipital areas, which are related to socioemotional and control functions (Aghajani et al., 2014; Wright et al., 2006). This possibility is supported by several DTI studies showing significant negative correlations between extraversion-related behaviors and the strength of fiber tracts, including IFOF, that interconnect frontal-temporal-occipital regions. A DTI study by Xu et al. (2012), for example, reported that higher scores on the fun-seeking subscale of the behavioral activation system were correlated with lower WM integrity (i.e., greater MD values) in the left IFOF and inferior longitudinal fasciculus. In a systematic review, Waller, Dotterer, Murray, Maxwell, & Hyde (2017) specify DTI studies that reported decreased FA in major WM fibers, including IFOF and forceps major, in adults with antisocial behaviors as compared to healthy controls. In another recent DTI study, Jiang et al. (2017) examined WM microstructure in antisocial personality disorder. They found decreased FA values in patients with antisocial disorder within the IFOF and other WM pathways, particularly those connecting the fronto-parietal control network and the fronto-temporal network. These pathways showed correlations with impulsivity and risky behaviors. The authors argued that damage to specific WM tracts might result in disinhibition, poor decision-making, and an inability to follow rules, due to impaired modulation of the frontal cortex.

Note that the findings reported by Waller et al. (2017) and Jiang et al. (2017) were based on studies of individuals with antisocial disorder, which is linked to psychoticism rather than to the extraversion dimension. Here, we found a similar pattern of decreased FA values in WM pathways, but it was associated with the extraversion dimension. This may indicate that characteristics related to the psychoticism dimension, such as impulsivity, are also related to extraversion. As a multifaceted concept, impulsivity, when broken down into its subfactors, may well occupy overlapping areas within the psychoticism and extraversion dimensions (Zuckerman & Glicksohn, 2016).

Overall, these studies suggest that decreased WM organization across regions and hemispheres is associated with behavioral characteristics that encompass both broad factors and narrower facets of extraversion. In accordance, extraverts typically show high functional connectivity within brain networks subserving reward and motivation in response to rewarding stimuli (Canli, 2004; Cohen, Young, Baek, Kessler, & Ranganath, 2005; Deckersbach et al., 2006). Similar findings have been reported in resting-state connectivity studies (Adelstein et al., 2011; Aghajani et al., 2014; Pang et al., 2016). For example, Pang et al. (2017) employed the functional connectivity density (FCD) method for the first time to distinguish the energy-efficient hubs associated with extraversion. Their results were extracted from resting-state fMRI data from 71 healthy participants and reported that short-range FCD was positively correlated with extraversion in the left cuneus, revealing a link between the local functional activity of this region and extraversion as expressed in risk-taking. In contrast, long-range FCD was negatively correlated with extraversion in the right superior frontal gyrus and the inferior frontal gyrus, a region uniquely positioned between emotional limbic regions and cognitive executive processing networks in the dorsal and medial areas of the PFC. Subsequent seed-based resting-state functional connectivity analyses revealed that a decreased long-range FCD in individuals with high extraversion scores showed a low long-range functional connectivity pattern between the medial and dorsolateral PFC, MTG, and anterior cingulate cortex. These regions are involved in inhibitory control and emotion perception and likely have an important role in extraversion (for details, see Pang et al., 2017).

It should be noted that characteristics and behaviors associated with extraversion, such as outgoing nature, novelty seeking, and reward sensitivity, can constitute both risk factors and protective factors (e.g., family and/or marital stressors, employment, environments, and genetics factors) and that the quality of these factors may depend on the strength of communication between them and the involved brain regions. However, we did not find increased FA in WM tracts interconnecting brain areas associated with regulatory control. Also, we did not find significant correlations between extraversion and WM tract integrity in other brain areas, for example, the uncinate fasciculus, longitudinal (arcuate) fasciculus, and insula, which were found in anatomical studies to be associated with extraversion (Aghajani et al., 2014; Gao et al., 2013; Pang et al., 2016). The most parsimonious interpretation is that these negative findings can be attributed to characteristics of the current sample. With no significant correlations between WM integrity and two personality traits (psychoticism and neuroticism), the present study supports Privado et al.’s (2017) assertion “that replicability is far from straightforward when structural brain features are related with the considered basic personality traits” (p.180).

Although DTI study in meditators is surprisingly rare, a few DTI studies have found a connection between meditation training and FA changes in tracts similar to those found in the current report, including increased FA of several fiber tracts, including the superior and anterior corona radiata, the genu and body of the CC, and the superior longitudinal fasciculus (Tang et al., 2009; Luders, Clark, Narr, & Toga, 2011). Also, specifically structured mindful sensorimotor training was found to increase FA in these tracts (Piervincenzi et al., 2017). Together, these results suggest that training, such as meditation and mindful sensorimotor training, might be a powerful tool to change the physical structure of the brain and thus of personality.

The current study focused on the association between trait extraversion and WM and did not examine the effect of training on the relationship between extraversion and WM. As such, EPQ traits were assessed after 12 weeks of training to avoid the effect of T1 measurement on trait values in T2 measurement. Although the prevailing assumption that personality traits are stable over time, personality traits may show mean level change across the life course (Caspi, Roberts, & Shiner, 2005; Harris, Brett, Johnson, & Deary, 2016). Since the correlation between FA, AD and RD and EPQ’s subscale scores was conducted only after 12 weeks of QMT training, and as training can affect the brain microstructure, the current results should be taken as suggestive and future studies should be conducted to examine this important connection between WM and personality.

Thus, an examination of possible effects of training on the level of trait extraversion in future research can expand the findings of the current work and also help to determine training-induced changes in personality and their underlying mechanism.

In conclusion, to better understand WM involvement in extraversion, we supplemented FA analysis with analyses of AD and RD, which helped provide more information about WM organization. All of the measures revealed associations between extraversion and the same WM tracts. We did not find correlations between AD and RD and other WM tracts, which were not detected by FA.

## 4. Limitations of the study

At present, we can report correlations, but not causal relationships, between WM organization (via FA, AD, and RD indices) and extraversion, such that the findings should be interpreted cautiously and future research should investigate the nature and implications of the current observations, by employing the effective connectivity method, which can provide information about causation (Sporns, 2013).

Furthermore, we used DTI images of 12 diffusion directions with four scan repetitions. Although more scan repetition seems to be related to a higher signal-to-noise ratio as well as to more reliable FA and tractography data (Jones, 2004; Farrell et al., 2007), it has been suggested that a larger number of diffusion gradient directions may improve the diffusion tensor estimation (Jones, 2004; Ni, Kavcic, Zhu, Ekholm, & Zhong, 2006). Therefore, future studies should include more directions of the diffusion gradient.

## 5. Conclusion

Our study revealed a link between extraversion and WM microstructure in a healthy sample, thereby providing insight into neurobiological mechanisms of extraversion. Evidence from functional and structural connectivity studies has shown that interindividual differences in extraversion and personality-related traits have biological bases in the brain. Whereas neuroscientists have begun to use DTI to examine the structural correlates of various psychiatric and neurological disorders (Kim & Whalen, 2009; Phan et al., 2009; Waller et al., 2017), few studies have examined the structural underpinnings of normal variations in personality characteristics using DTI and even fewer have examined extraversion as a trait (Xu & Potenza, 2012). To our knowledge, there was no previous investigation of the association between extraversion and FA, AD, and RD indices.

Given the role of extraversion in affect and in the development of both adaptive and risky behaviors, further knowledge about the relationship between WM integrity and extraversion may have broad applicability. In particular, it would be interesting to examine the effect of extraversion on risky behaviors (smoking, alcohol use, and violence) as compared to adaptive behaviors in different populations and to further research the neural underpinnings of behavioral differences as a function of extraversion.
